# Isolation and Characterization of Phenolic Antioxidants from Plantago Herb

**DOI:** 10.3390/molecules17055459

**Published:** 2012-05-09

**Authors:** Yoshiaki Amakura, Ayako Yoshimura, Morio Yoshimura, Takashi Yoshida

**Affiliations:** College of Pharmaceutical Sciences, Matsuyama University, 4-2 Bunkyo-cho, Matsuyama, Ehime 790-8578, Japan; Email: 16061597@cc.matsuyama-u.ac.jp (A.Y.); myoshimu@cc.matsuyama-u.ac.jp (M.Y.); tyoshida@gem.e-catv.ne.jp (T.Y.)

**Keywords:** *Plantago asiatica*, Plantaginaceae, phenylethanoid glycoside, antioxidant, oxygen radical absorbance capacity (ORAC)

## Abstract

Seven phenolic compounds, including a new phenylethanoid glycoside, were isolated from the ethyl acetate fraction of an aqueous ethanol extract of Plantago Herb (whole part of *Plantago asiatica* L.), which showed significant antioxidative activity. The new compound was characterized as 2-(3,4-dihydroxyphenyl)ethyl 3-*O*-β-D-allopyranosyl-6-*O*-caffeoyl-β-D-glucopyranoside on the basis of spectral and chemical evidence, and its antioxidant activity was comparable to that of tea catechins.

## 1. Introduction

The genus *Plantago* consists of more than 200 species, most of which are small plants with elliptic leaves and small spikes of very small flowers. *Plantago asiatica* L. (Plantaginaceae) is a weed widely distributed in eastern Asia. The aerial parts of *P. asiatica*, ‘Plantago Herba’, are used as a crude drug in China, Korea, and Japan for diuretic, antitussive, expectorant, and antiphlogistic purposes [1]. In Japan, it is an official medicine listed in the Japanese Pharmacopeia as the crude drug “Plantago Herb” [2]. It has also been commercialized as a dietary supplement in various forms, such as a tea. Polyphenols such as phenylethanoid glycosides and flavonoids, together with iridoid glucosides, were reported as components of the aerial parts of this plant [3]. Plantamajoside, a phenylethanoid of the major constituent of this plant, has been reported to exhibit antibacterial, antiallergic, anti-inflammatory, antioxidant, and enzyme inhibitory activities [3]. Thus, it is regarded that Plantago Herb is a plant material rich in polyphenolics beneficial to human health. We have investigated the polyphenolics of medicinal plants and foods, and reported the characteristic polyphenols such as flavonoids, tannins, and related polyphenols in herbs, spices, and medicinal plants [4,5,6,7]. As part of our investigation, we herein report the isolation and characterization of phenolic compounds in the active extracts from Plantago Herb based on antioxidant assay-guided fractionation and purification.

## 2. Results and Discussion

**Figure 1:**
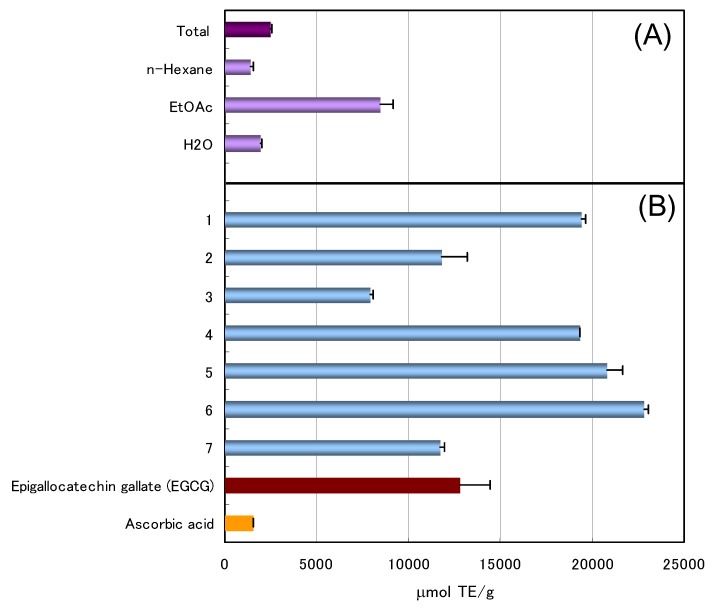
ORAC values of each fraction (**A**) and isolated compounds (**B**).

**Figure 2:**
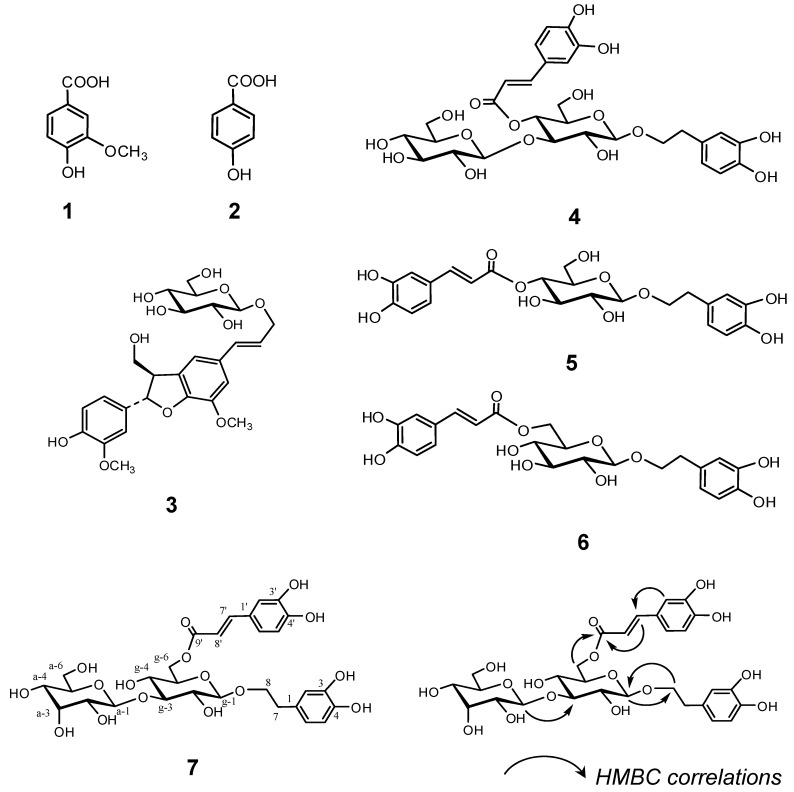
Structures of compounds **1**–**7** and selected HMBC correlations of **7**.

The Plantago Herb was homogenized in 80% EtOH and the homogenate was filtered. The filtrate was concentrated and extracted with *n*-hexane and ethyl acetate (EtOAc), to give the respective *n*-hexane, EtOAc, and water extracts. The antioxidative activity of each extract was evaluated on the basis of the oxygen radical absorbance capacity (ORAC) [8,9] (Figure 1A). The EtOAc extract, which exhibited marked antioxidative activity, was chromatographed over MCI-GEL CHP-20P with MeOH-H_2_O in a stepwise gradient mode. The fractions showing similar HPLC patterns were combined and further purified by column chromatography over Sephadex LH-20 with EtOH and/or YMC GEL ODS-AQ with aqueous MeOH, to afford vanillic acid (**1**) [10], *p*-hydroxybenzoic acid (**2**) [11], (*7S*, *8R*)-dehydrodiconiferyl alcohol 9'-β-D-glucopyranoside (**3**) [12], plantamajoside (**4**) [13], desrhamnosyl acteoside (**5**) [14], and calceorioside B (**6**) [14] together with a new phenylethyl glycoside, compound **7** (Figure 2). The known compounds **1**–**6** were identified by direct comparison with authentic specimens and by comparison of their spectral data with those reported in the literature.

Compound **7**, was isolated as a brown amorphous powder. Its molecular formula was assigned as C_29_H_36_O_16_ from its HR-ESI-MS (*m/z* 639.1939 [M−H]^−^; calcd. for C_29_H_36_O_16_-H: 639.1931) and ^13^C-NMR (29 ^13^C signals) spectra. The UV spectrum showed λ_max_ MeOH nm (log ε) at 204 (4.31), 218sh (4.17), 247sh (3.89), 289 (4.00), and 328 (4.10). The ^1^H- and ^13^C-NMR spectral data of **7** exhibited the characteristic signals of *trans*-caffeoyl and 3,4-dihydroxyphenethyl alcohol moieties, as shown in Table 1. The presence of two sugar units was also suggested by two distinctive anomeric signals in the spectra. The NMR data were fully assigned by 1D and 2D spectra referring to those of **4** with similar units. D-Glucose and D-allose were confirmed as the sugar units in **7** according to a previously described method [15], as follows: compound **7** (1.0 mg) was hydrolyzed by heating in 0.5 M HCl, followed by neutralization with Amberlite IRA400. After drying, the residue was dissolved in pyridine containing L-cysteine methyl ester hydrochloride and heated at 60 °C for 1 h. *o*-Tolyl isothyocyanate in pyridine was then added to the mixture and further heated at 60 °C for 1 h. The reaction mixture was directly analyzed by RP-HPLC to detect peaks identical with those of authentic derivatives prepared by a similar reaction of D-allose and D-glucose. The linking position of each unit was confirmed by cross-peaks between glucose H-1 (δ 4.38) and C-8 (δ 72.4) of 3,4-dihydroxyphenylethyl alcohol, glucose H-6 (δ 4.51, 4.33) and C-9' (δ 169.1) of caffeoyl group, and allose H-1 (δ 4.87) and glucose C-3 (δ 88.1) (Figure 2) in HMBC. β-Glycosidic linkages were evidenced by large coupling constants (*J* = 8 Hz). Therefore, compound **7** was established as 2-(3,4-dihydroxyphenyl)ethyl 3-*O*-β-D-allopyranosyl-6-*O*-caffeoyl-β-D-glucopyranoside.

**Table 1:** ^1^H- (500 MHz) and ^13^C-NMR (126 MHz) data of compound **7** measured in MeOH-*d*_4_.

Position	δC	δH ( *J* in Hz)
1	131.4	
2	117.1	6.66 (d, *J* = 2)
3	144.7	
4	146.1	
5	121.3	6.62 (d, *J* = 8)
6	116.5	6.53 (dd, *J* = 2, 8)
7	36.7	2.78 (m)
8	72.4	3.75, 3.95 (each m)
1'	127.7	
2'	115.7	7.02 (d, *J* = 2)
3'	146.1	
4'	149.1	
5'	116.5	6.76 (d, *J* = 8)
6'	123.2	6.88 (dd, *J* = 2, 8)
7'	147.2	7.55 (d, *J* = 16)
8'	114.8	6.25 (d, *J* = 16)
9'	169.1	
glucose-1	104.1	4.38 ( *J* = 8)
2	74.3	3.20–3.60 ^a^
3	88.1	3.20–3.60 ^a^
4	70.4	3.20–3.60 ^a^
5	75.1	3.20–3.60 ^a^
6	64.6	4.51 (dd, *J* = 2, 12), 4.33 (dd, *J* = 6, 12)
allose-1	103.1	4.87 (d, *J* = 8)
2	72.8	3.41–3.49 ^b^
3	72.9	4.06 (d, *J* = 3)
4	68.9	3.41–3.49 ^b^
5	75.9	3.70 (m)
6	63.0	3.62 (dd, *J* = 6, 11.5), 3.85 (dd, *J* = 2, 11.5)

^a, b^ Overlapped signals.

The antioxidant activity of isolated compounds **1**–**7** was estimated based on ORAC values (Figure 1B). Compounds **1**, **4**, **5**, and **6** showed potent antioxidative activity with ORAC values of *ca*. 20,000 μmol TE/g, which were roughly two times more potent than epigallocatechin gallate (EGCG), a typical tea catechin. The potency of compound **7** was comparable to that of EGCG. The marked activity of the EtOAc extract is considered to be responsible for **4** being the main component in the extract. Thus, **4** might be useful as a marker of the antioxidant activity of Plantago Herb. These results also suggested that the antioxidant activity of Plantago Herb can largely be attributed to these isolated phenolic compounds.

## 3. Experimental

### 3.1. General

Optical rotations were measured with a JASCO P-1020 digital polarimeter. UV spectra were recorded on a Shimadzu UVmini-1240 (Kyoto, Japan). Electrospray ionization (ESI)-MS, and high-resolution (HR) ESI-MS spectra were obtained using a micrOTOF-Q (Bruker Daltonics, Billerica, MA, USA) mass spectrometer using acetonitrile as the solvent. ^1^H- and ^13^C-NMR spectra were recorded on a Brucker AVANCE500 instrument (Bruker BioSpin, Billerica, MA, USA) (500 MHz for ^1^H and 126 MHz for ^13^C) and chemical shifts are given in ppm values relative to those of the solvents [methanol-*d*_4_ (δ_H_ 3.30; δ_C_ 49.0)] on a tetramethylsilane scale. The standard pulse sequences programmed for the instrument (AVANCE 500) were used for each 2D measurement (COSY, HSQC, and HMBC). *J*_CH_ was set at 8 or 10 Hz in HMBC. Column chromatography was carried out with Diaion HP-20, MCI-gel CHP-20P (Mitsubishi Chemical Co., Tokyo, Japan), YMC gel ODS (YMC Co. Ltd., Kyoto, Japan), and Sephadex LH-20 (GE Healthcare, Little Chalfont, UK), respectively. Normal-phase (NP) HPLC conducted on a YMC-Pack SIL A-003 (YMC Co., Ltd.) column (4.6 i.d. × 150 mm) developed with *n*-hexane-MeOH-tetrahydrofuran-formic acid (55:33:11:1) containing oxalic acid (450 mg/L) (flow rate: 1.5 mL/min; 280 nm UV detection). Reversed-phase (RP) HPLC conditions were as follows: (Condition 1) column, L-column ODS (5 μm, 150 × 2.1 mm i.d.) (Chemicals Evaluation and Research Institute, Tokyo, Japan); mobile phase, solvent A was 5% acetic acid and solvent B was acetonitrile (0–30 min, 0–50% B in A; 30–35 min, 50–85% B in A; 35–40 min, 85–85% B in A); injection volume, 2 μL; column temperature, 40 °C; flow-rate, 0.3 mL/min; detection, 200–400 nm. (Condition 2) column, YMC-Pack ODS AQ (5 μm, 150 × 2.0 mm i.d.) (YMC Co. Ltd.); mobile phase, 10 mM H_3_PO_4_-10 mM KH_2_PO_4_-acetonitrile (41:41:18); column temperature, 40 °C; flow-rate, 0.2 mL/min; detection, 280 nm. (Condition 3) column, COSMOSIL Cholester Waters (5 μm, 150 × 2.0 mm i.d.) (nacalai tesque, Kyoto, Japan); mobile phase, 10 mM H_3_PO_4_-10 mM KH_2_PO_4_-MeOH (37.5:37.5:25); column temperature, 40 °C; flow-rate, 0.2 mL/min; detection, 280 nm. Microplate reader was used an Infinite F200 microplate reader (TECAN, Männedorf, Switzerland).

### 3.2. Samples and Reagents

Plantago Herb (dried whole plant of *Plantago asiatica* L) was obtained from Uchida Wakanyaku Ltd. (Tokyo, Japan). *N*-Methylmorpholine-*N*-oxide, 2,2'-azobis-(2-amidinopropane) dihydrochloride (AAPH), L-cysteine methyl ester hydrochloride and *o*-tolyl isothiocyanate were purchased from Wako Pure Chemical Industries (Osaka, Japan). Fluorescein sodium salt and 6-hydroxy-2,5,7,8-tetramethylchroman-2-carboxylic acid (Trolox) were obtained from Sigma-Aldrich (St. Louis, MO, USA). All other reagents were of analytical grade. 

### 3.3. Extraction and Isolation

The Plantago Herb (500 g) was homogenized in 80% EtOH [EtOH-H_2_O (8:2)] (5 L) and the homogenate was filtered. The filtrate was concentrated and extracted with *n*-hexane (0.6 L) and ethyl acetate (1.2 L), to give the respective *n*-hexane (338.8 mg), ethyl acetate (8.6 g), and water (60.7 g) extracts. The ethyl acetate extract (4 g) was chromatographed over MCI-GEL CHP-20P with MeOH-H_2_O (0:100→10:90→20:80→30:70→40:60→100:0) in stepwise gradient mode. The fractions showing similar HPLC patterns were combined and further purified by column chromatography over Sephadex LH-20 with EtOH and/or YMC GEL ODS-AQ with aqueous MeOH to afford vanillic acid (**1**) (26.1 mg), *p*-hydroxybenzoic acid (**2**) (2.1 mg), (*7S*, *8R*)-dehydrodiconiferyl alcohol 9'-β-D-glucopyranoside (**3**) (7.9 mg), plantamajoside (**4**) (1.16 g), desrhamnosyl acteoside (**5**) (29.0 mg), calceorioside B (**6**) (109.3 mg), 2-(3,4-dihydroxyphenyl)ethyl 3-*O*-β-D-allopyranosyl-6-*O*-caffeoyl-β-D-glucopyranoside (**7**) (29.4 mg). On the other hand, the water extract (60 g) was separated by column chromatography over Diaion HP-20 with aqueous MeOH to give **4** (6.6 g). These compounds were identified by direct comparison with authentic specimens or by comparison of their spectral data with those reported in the literature. The physical data of new compound **7** are as follows.

*2-(3*, *4-Dihydroxyphenyl)ethyl 3-O- β- D-allopyranosyl-6-O-caffeoyl- β- D- glucopyranoside* (**7**): UV λ_max_ (MeOH) nm (ε): 204 (4.31), 218sh (4.17), 247sh (3.89), 289 (4.00), 328 (4.10). [α]^23^_D_ −22.1° (*c* 1.8, MeOH). ^1^H-NMR (500 MHz, methanol-*d*_4_) δ_H_: 7.55 (1H, d, *J* = 16 Hz, H-7'), 7.02 (1H, d, *J* = 2 Hz, H-2'), 6.88 (1H, dd, *J* = 2, 8 Hz, H-6'), 6.76 (1H, d, *J* = 8 Hz, H-5'), 6.66 (1H, d, *J* = 2 Hz, H-2), 6.62 (1H, d, *J* = 8 Hz, H-5), 6.53 (1H, dd, *J* = 2, 8 Hz, H-6), 6.25 (1H, d, *J* = 16 Hz, H-8'), 4.87 (1H, d, *J* = 8 Hz, all H-1), 4.51 (1H, dd, *J* = 2, 12, glc H-6a), 4.38 (1H, d, *J* = 8 Hz, glc H-1), 4.33 (1H, dd, *J* = 6, 12 Hz, glc H-6b), 4.06 (1H, d, *J* = 3 Hz, all H-3), 3.95 (1H, m, H-8a), 3.85 (1H, dd, *J* = 2, 11.5 Hz, all H-6a), 3.76 (1H, m, all H-5), 3.62 (1H, dd, *J* = 6, 11.5 Hz, all H-6b), 3.20–3.60 (4H, m, glc H-2-5), 3.41–3.49 (2H, m, all H-2, 4), 2.78 (2H, m, H-7). ^13^C-NMR δ_C_: 36.7 (C-7), 63.0 (all C-6), 64.6 (glc C-6), 68.9 (all C-4), 70.4 (glc C-4), 72.4 (C-8), 72.8 (all C-2), 72.9 (all C-3), 74.3 (glc C-2), 75.1 (glc C-5), 75.9 (all C-5), 88.1 (glc C-3), 103.1 (all C-1), 104.1 (glc C-1), 114.8 (C-8'), 115.7 (C-2'), 116.5 (2C, C-5, 5'), 117.1 (C-2), 121.3 (C-6), 123.2 (C-6'), 127.7 (C-1'), 131.4 (C-1), 144.7 (C-3), 146.1 (2C, C-4, 3'), 147.2 (C-7'), 149.1 (C-4'), 169.1 (C-9'). HR-ESI-MS *m/z*: 639.1939 ([M−H]^−^, Calcd. for C_29_H_36_O_16_-H: 639.1931).

### 3.4. Determination of Sugar Configuration

Sugar configuration was determined using previous described methods. Compound **7** (1.0 mg) were hydrolyzed by heated in 0.5 M HCl (0.2 mL) and neutralized with Amberlite IRA400. After drying, the residue was dissolved in pyridine (0.2 mL) containing L-cysteine methyl ester hydrochloride (1.0 mg) and heated at 60 °C for 1 h. After heating, *o*-tolyl isothyocyanate (1.0 mg) in pyridine (0.2 mL) was added to the mixture and heated at 60 °C for 1h. The reaction mixture was directly analyzed by RP-HPLC. The peaks were coincided with derivatives of D-allose and D-glucose.

### 3.5. Antioxidant Assay

Antioxidant activity was estimated by the ORAC method. Measurement of the ORAC values was performed using previous described methods. Briefly, the ORAC assay was performed in 75 mM phosphate buffer (pH 7.4) with a final reaction volume of 200 μL. Trolox (20 μL) and fluorescein (120 μL; 70 nM, final concentration) solutions were pipetted into each well of a 96-well microplate. The mixture was pre-incubated in a microplate reader for 15 min at 37 °C. A solution of AAPH (60 μL: final concentration, 12 mM) was added rapidly to the microplate, and after shaking for 15 s, the fluorescence was recorded every minute for 90 min at excitation and emission wavelengths of 485 and 528 nm, respectively. The area under the curve (AUC) was calculated, and the net AUC was calculated by subtracting the AUC of the blank (phosphate buffer only) from that of each sample. ORAC values were expressed as trolox equivalents (μmol TE/g) using the calibration curve generated in each assay.

## 4. Conclusions

The new phenylethanoid glycoside, 2-(3,4-dihydroxyphenyl)ethyl-3-*O*-β-D-allopyranosyl-6-*O*-caffeoyl-β-D-glucopyranoside (**7**), was isolated from Plantago Herb (whole part of *Plantago asiatica* L.), together with six known phenolic compounds. Among them, vanillic acid (**1**), plantamajoside (**4**), desrhamnosyl acteoside (**5**), and calceorioside B (**6**) showed potent antioxidant activity. The potency of **7** was comparable to that of EGCG. 

## Supplementary Materials

Supplementary materials can be accessed at: http://www.mdpi.com/1420-3049/17/5/5459/s1.
