# Supplementary material for: Isolation and Characterization of Phenolic Antioxidants from Plantago Herb
Source: Molecules. 2012 May 9;17(5):5459–66. doi: 10.3390/molecules17055459 (PMC6268151; doi:10.3390/molecules17055459)
Supplement: Supplementary file 1 [file molecules-17-05459-s001.pdf]

^{1}H-NMR

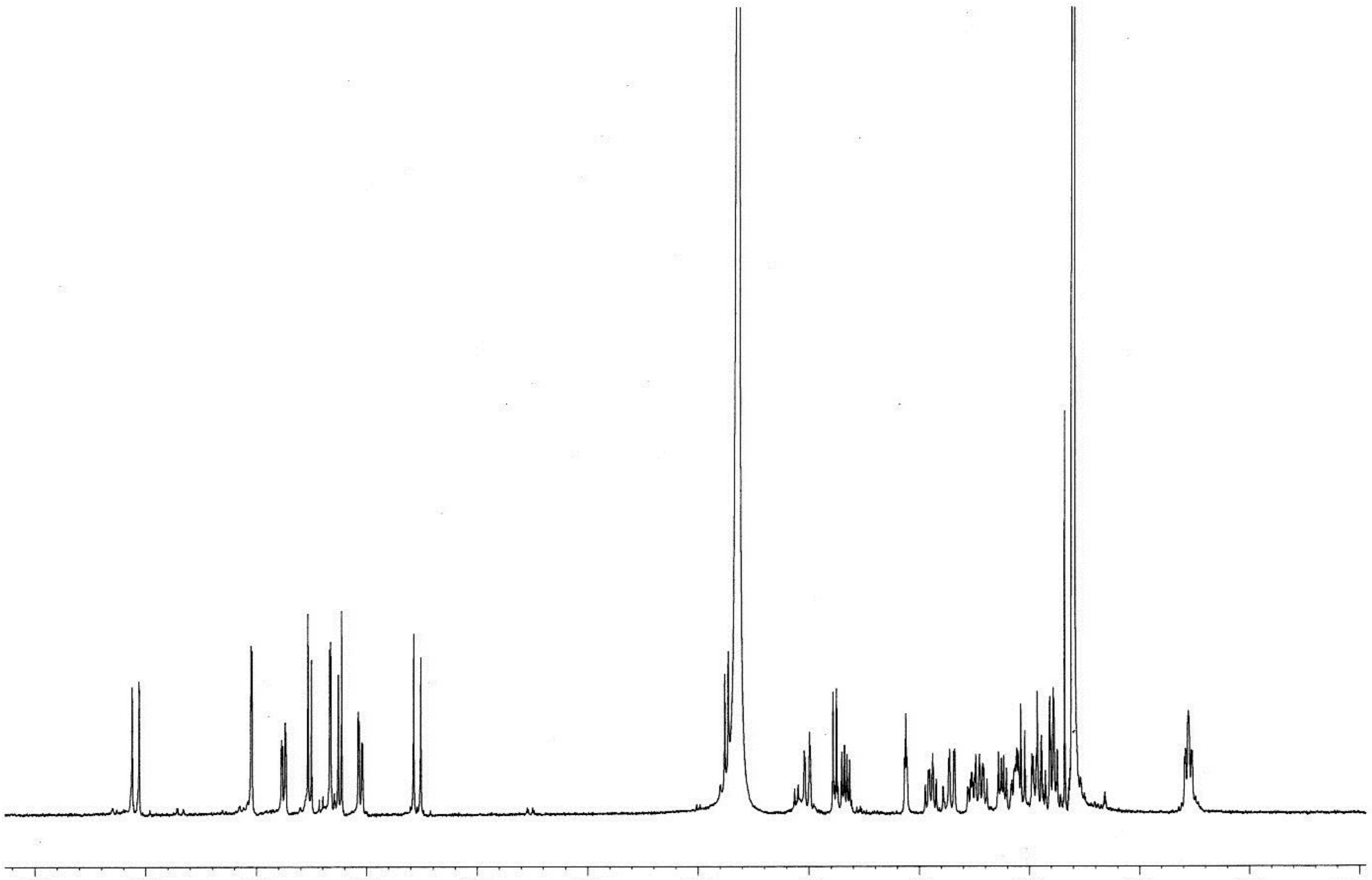


```
Current Data Parameters
NAME             Amakura
EXPNO                640
PROCNO                 1

F2 - Acquisition Parameters
Date_           20100710
Time               18.25
INSTRUM            spect
PROBHD   5 mm PABBO BB-
PULPROG             zg30
TD                 32768
SOLVENT             MeOD
NS                   256
DS                     0
SWH            10330.578 Hz
FIDRES          0.315264 Hz
AQ             1.5860212 sec
RG                  1290
DW                48.400 usec
DE                  6.00 usec
TE                 300.0 K
D1            0.50000000 sec
TD0                    1

======== CHANNEL f1 ========
NUC1                  1H
P1                  2.00 usec
PL1                 3.00 dB
SFO1         500.1330885 MHz

F1 - Acquisition parameters
ND0                    1
TD                   128
SFO1            500.1331 MHz
FIDRES          7.812500 Hz
SW                 1.999 ppm
FnMODE                QF

F2 - Processing parameters
SI                 32768
SF           500.1300158 MHz
WDW                   EM
SSB                    0
LB                  0.30 Hz
GB                     0
PC                  1.00

F1 - Processing parameters
SI                  1024
MC2                   QF
SF           500.1300000 MHz
WDW                 SINE
SSB                    0
LB                  0.30 Hz
GB                   0.1
```

^{13}C-NMR

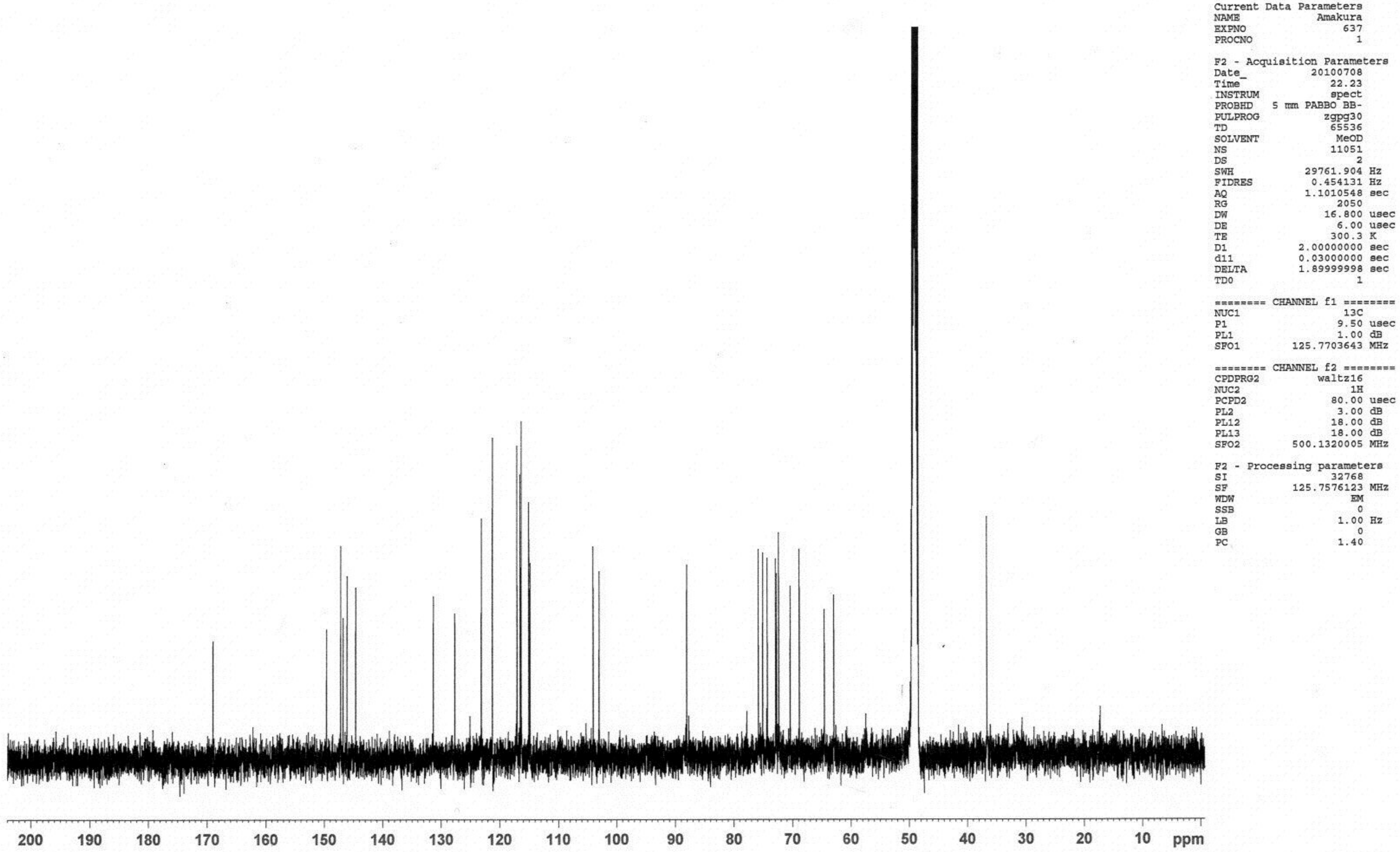
Current Data Parameters
NAME Amakura
EXPNO 637
PROCNO 1
F2 - Acquisition Parameters
Date_ 20100708
Time 22.23
INSTRUM spect
PROBHD 5 mm PABBO BB-
PULPROG zgpg30
TD 65536
SOLVENT MeOD
NS 11051
DS 2
SWH 29761.904 Hz
FIDRES 0.454131 Hz
AQ 1.1010548 sec
RG 2050
DW 16.800 usec
DE 6.00 usec
TE 300.3 K
D1 2.00000000 sec
d11 0.03000000 sec
DELTA 1.89999998 sec
TD0 1
======== CHANNEL f1 ========
NUC1 13C
P1 9.50 usec
PL1 1.00 dB
SFO1 125.7703643 MHz
======== CHANNEL f2 ========
CPDPRG2 waltz16
NUC2 1H
PCPD2 80.00 usec
PL2 3.00 dB
PL12 18.00 dB
PL13 18.00 dB
SFO2 500.1320005 MHz
F2 - Processing parameters
SI 32768
SF 125.7576123 MHz
WDW EM
SSB 0
LB 1.00 Hz
GB 0
PC 1.40
200 190 180 170 160 150 140 130 120 110 100 90 80 70 60 50 40 30 20 10 ppm

^1H-^1H COSY

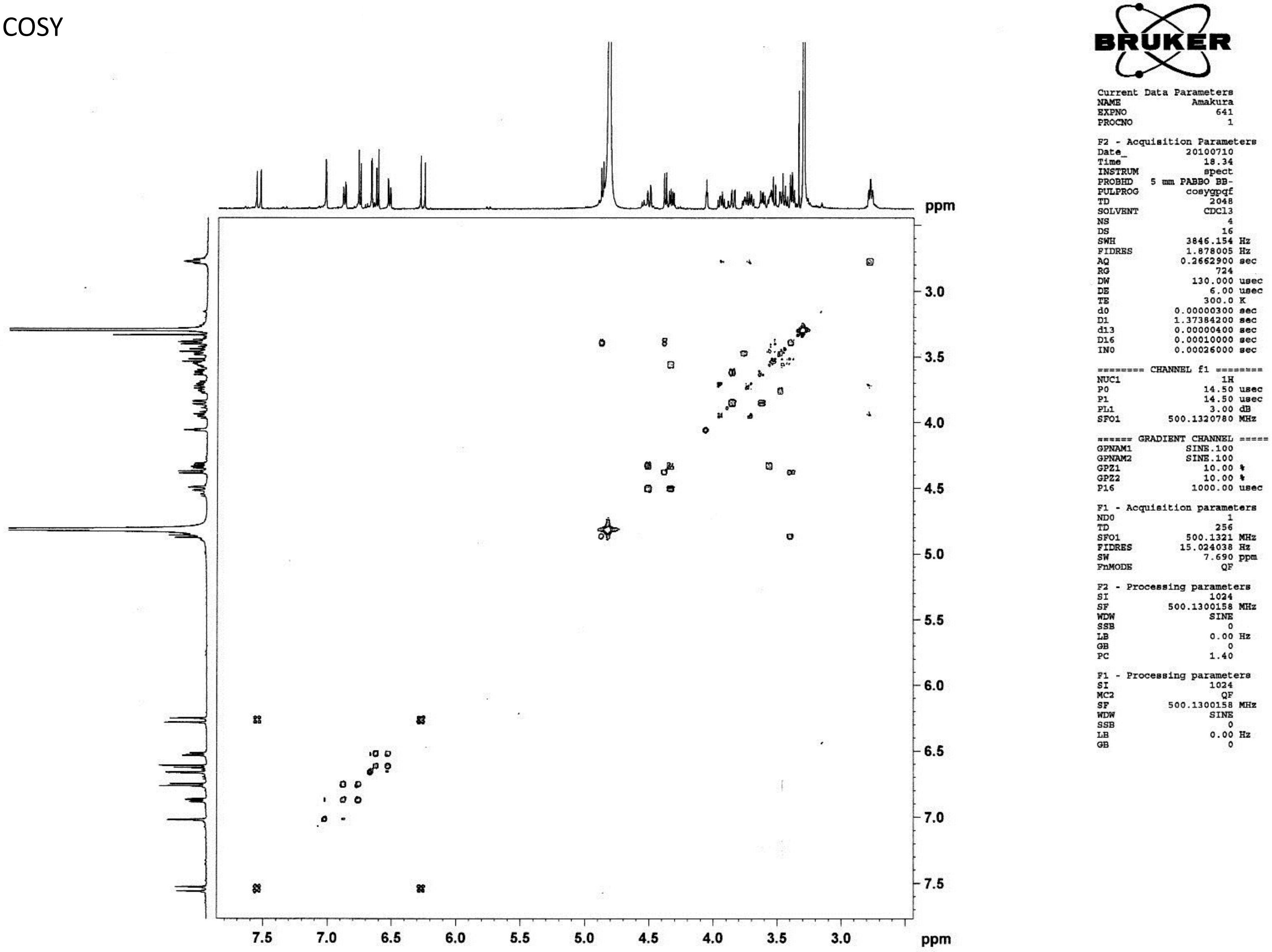
ppm
3.0
3.5
4.0
4.5
5.0
5.5
6.0
6.5
7.0
7.5
7.5
7.0
6.5
6.0
5.5
5.0
4.5
4.0
3.5
3.0
ppm
Current Data Parameters
NAME Amakura
EXPNO 641
PROCNO 1
F2 - Acquisition Parameters
Date_ 20100710
Time 18.34
INSTRUM spect
PROBHD 5 mm PABBO BB-
PULPROG cosygpqf
TD 2048
SOLVENT CDCl3
NS 4
DS 16
SWH 3846.154 Hz
FIDRES 1.878005 Hz
AQ 0.2662900 sec
RG 724
DW 130.000 usec
DE 6.00 usec
TE 300.0 K
d0 0.00000300 sec
D1 1.37384200 sec
d13 0.00000400 sec
D16 0.00010000 sec
IN0 0.00026000 sec
======== CHANNEL f1 ========
NUC1 1H
P0 14.50 usec
P1 14.50 usec
PL1 3.00 dB
SFO1 500.1320780 MHz
====== GRADIENT CHANNEL =====
GPNAM1 SINE.100
GPNAM2 SINE.100
GPZ1 10.00 %
GPZ2 10.00 %
P16 1000.00 usec
F1 - Acquisition parameters
ND0 1
TD 256
SFO1 500.1321 MHz
FIDRES 15.024038 Hz
SW 7.690 ppm
FnMODE QF
F2 - Processing parameters
SI 1024
SF 500.1300158 MHz
WDW SINE
SSB 0
LB 0.00 Hz
GB 0
PC 1.40
F1 - Processing parameters
SI 1024
MC2 QF
SF 500.1300158 MHz
WDW SINE
SSB 0
LB 0.00 Hz
GB 0

HSQC

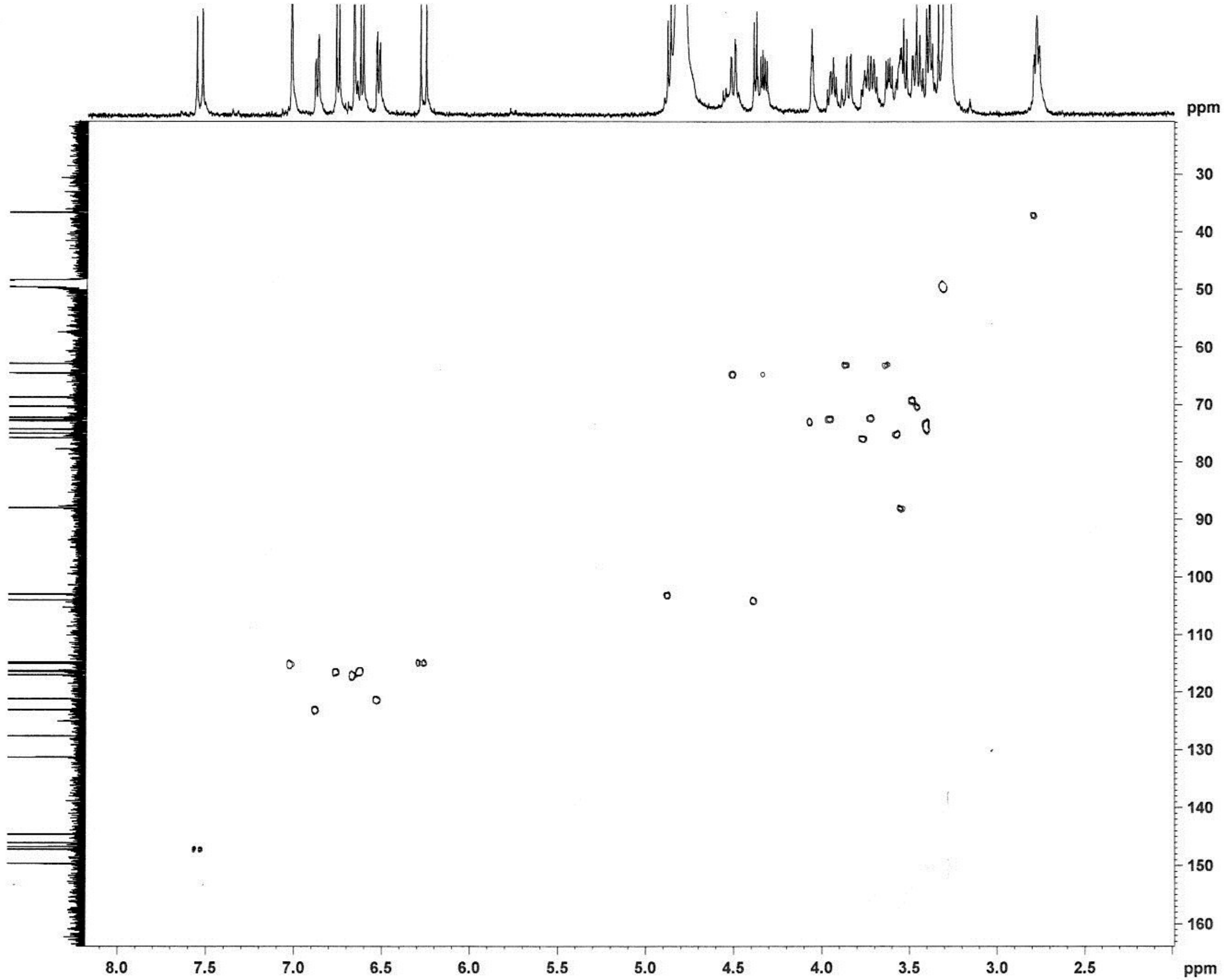


```
Current Data Parameters
NAME              Amakura
EXPNO                 638
PROCNO                  1

F2 - Acquisition Parameters
Date_            20100709
Time                 8.07
INSTRUM             spect
PROBHD   5 mm PABBO BB-
PULPROG        hsqcedetgp
TD                   1024
SOLVENT              MeOD
NS                      4
DS                     16
SWH              4065.041 Hz
FIDRES           3.969766 Hz
AQ              0.1260020 sec
RG                   2050
DW                123.000 usec
DE                   6.00 usec
TE                  299.9 K
CNST2         145.0000000
d0             0.00000300 sec
D1             1.45064294 sec
d4             0.00172414 sec
d11            0.03000000 sec
d13            0.00000400 sec
D16            0.00020000 sec
D21            0.00345000 sec
DELTA          0.00221500 sec
DELTA1         0.00071614 sec
IN0            0.00002000 sec
ST1CNT                128
ZGOPTNS

======== CHANNEL f1 ========
NUC1                   1H
P1                  14.50 usec
p2                  29.00 usec
P28                  0.00 usec
PL1                  3.00 dB
SFO1          500.1322476 MHz

======== CHANNEL f2 ========
CPDPRG2              garp
NUC2                  13C
P3                   9.80 usec
p4                  19.60 usec
PCPD2               65.00 usec
PL2                  1.40 dB
PL12                17.83 dB
SFO2          125.7710857 MHz

====== GRADIENT CHANNEL =====
GPNAM1           SINE.100
GPNAM2           SINE.100
GPZ1                80.00 %
GPZ2                20.10 %
P16               1000.00 usec

F1 - Acquisition parameters
ND0                     2
TD                    172
SFO1             125.7711 MHz
FIDRES         145.348831 Hz
SW                198.774 ppm
FnMODE     Echo-Antiecho

F2 - Processing parameters
SI                   1024
SF            500.1300104 MHz
WDW                 QSINE
SSB                     2
LB                   0.00 Hz
GB                      0
PC                   1.40

F1 - Processing parameters
SI                   1024
MC2        echo-antiecho
SF            125.7575861 MHz
WDW                 QSINE
SSB                     2
LB                   0.00 Hz
GB                      0
```

HMBC

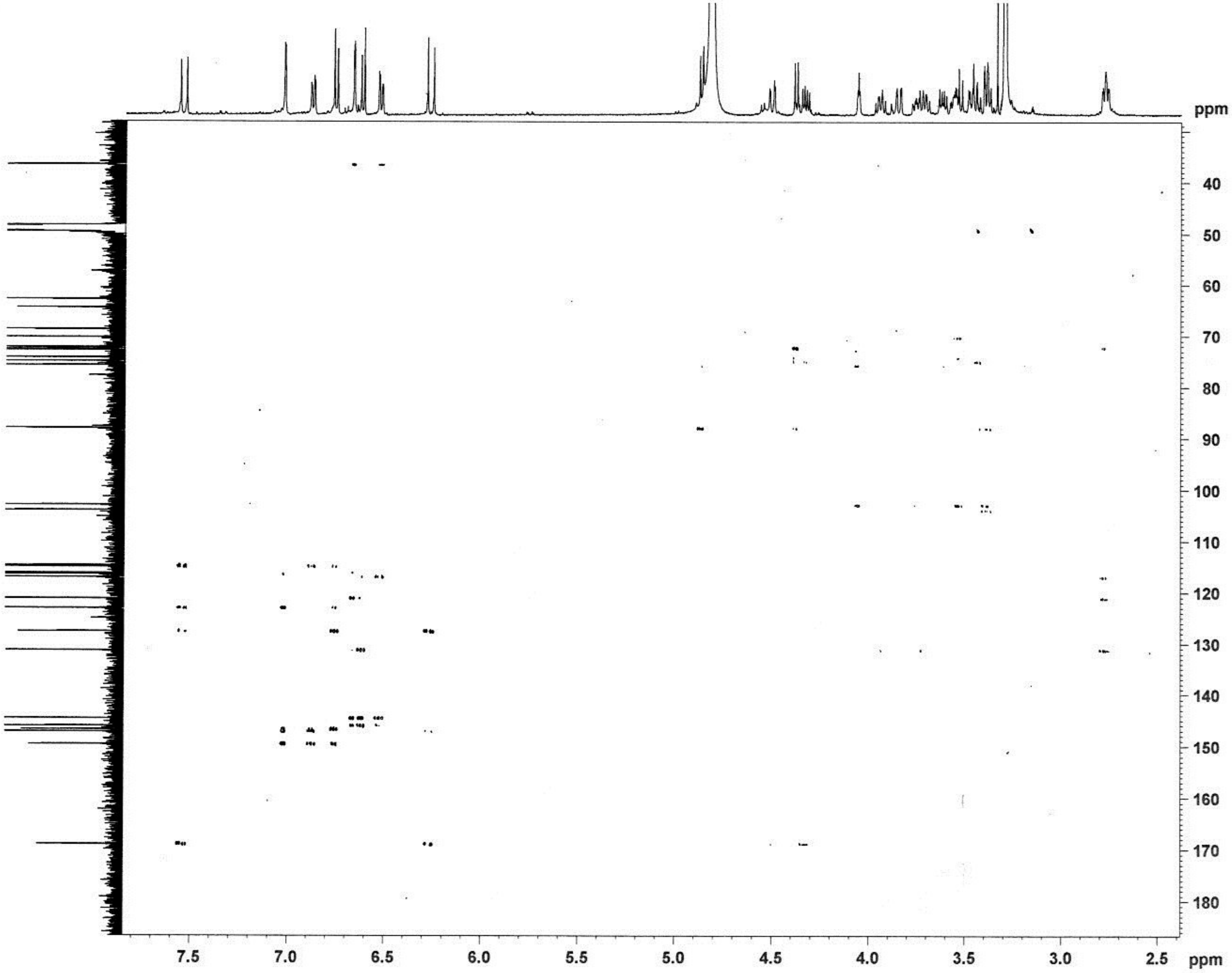


```
Current Data Parameters
NAME              Amakura
EXPNO                 642
PROCNO                  1

F2 - Acquisition Parameters
Date_            20100710
Time                19.08
INSTRUM             spect
PROBHD   5 mm PABBO BB-
PULPROG      hmbcgplpndqf
TD                   4096
SOLVENT              MeOD
NS                     16
DS                     16
SWH              2976.190 Hz
FIDRES           0.726609 Hz
AQ              0.6881780 sec
RG                   2050
DW                168.000 usec
DE                   6.00 usec
TE                  300.0 K
CNST2         145.0000000
CNST13          8.0000000
d0             0.00000300 sec
D1             1.12644303 sec
d2             0.00344828 sec
d6             0.06250000 sec
D16            0.00010000 sec
IN0            0.00002000 sec

======== CHANNEL f1 ========
NUC1                   1H
P1                  14.50 usec
p2                  29.00 usec
PL1                  3.00 dB
SFO1          500.1325634 MHz

======== CHANNEL f2 ========
NUC2                  13C
P3                   9.80 usec
PL2                  1.40 dB
SFO2          125.7710857 MHz

====== GRADIENT CHANNEL =====
GPNAM1           SINE.100
GPNAM2           SINE.100
GPNAM3           SINE.100
GPZ1                50.00 %
GPZ2                30.00 %
GPZ3                40.10 %
P16               1500.00 usec

F1 - Acquisition parameters
ND0                     2
TD                   1495
SFO1             125.7711 MHz
FIDRES          16.722408 Hz
SW                198.774 ppm
FnMODE                 QF

F2 - Processing parameters
SI                   2048
SF            500.1300158 MHz
WDW                  SINE
SSB                     0
LB                   0.00 Hz
GB                      0
PC                   1.40

F1 - Processing parameters
SI                   1024
MC2                    QF
SF            125.7576123 MHz
WDW                  SINE
SSB                     0
LB                   0.00 Hz
GB                      0
```
